# 

**Published:** 1896-04

**Authors:** 


APRIL, 1896.

### THE AMERICAN JOURNAL OF DENTAL SCIENCE.

EDITED BY

F. J. S. GORGAS, M. D., D. D. S.

RICHARD GRADY, M. D., D. D. S.

Vol. 29. THIRD SERIES No. 12.

PUBLISHED MONTHLY.

$2.50 PER ANNUM.

BALTIMORE:

SNOWDEN & COWMAN MFG. CO., 9 W. Fayette St.

LONDON:
TRUBNER & CO., 60 Paternoster Row.

Entered at the Post Office at Baltimore, Md., as Second-class Matter.

PAYABLE IN ADVANCE.

